# Combined Spinal Epidural Anaesthesia for Gastroschisis Repair

**Published:** 2009-04

**Authors:** Mangesh Gore, Kunal Joshi, Nandini Dave

**Affiliations:** 1 Lecturer, Department of Anaesthesiology, T N Medical College & B Y L Nair Hospital, Mumbai; 2 P.G.Student, Department of Anaesthesiology, T N Medical College & B Y L Nair Hospital, Mumbai; 3 Associate Professor, Department of Anaesthesiology, T N Medical College & B Y L Nair Hospital, Mumbai

**Keywords:** Gastroschisis, Combined spinal epidural

## Abstract

**Summary:**

Gastroschisis is a congenital anomaly with a high perioperative mortality. Administration of general anaesthesia to these high risk neonates is associated with several problems including postoperative apnoea and the need for mechanical ventilation. Central neuraxial blocks, and more recently, combined spinal epidural have been administered for major abdominal surgery in neonates. We present the case of a neonate posted for gastroschisis repair conducted under combined spinal epidural anaesthesia and discuss the several advantages of this technique.

## Introduction

Gastroschisis is a congenital anomaly with abdominal wall defect with protrusion of abdominal viscera outside the abdominal cavity. The incidence is 2-4.9 per 10000 live births with a male preponderance^1^. Administration of general anaesthesia to this group of patients is associated with several problems including the need for postoperative ventilation. Central neuraxial block for gastroschisis repair in neonates is an alternative technique which has proven to be safe with minimal complications^2^–^4^. We report a case of a neonate with gastroschisis operated under combined spinal- epidural block and discuss the advantages of the technique.

## Case report

**Fig 1:**
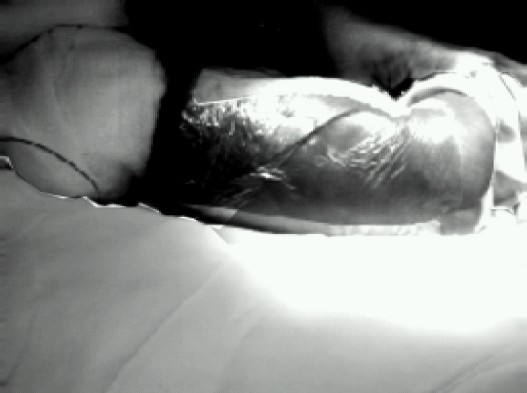
Epidural catheter tunneled away from insertion site

**Fig 2:**
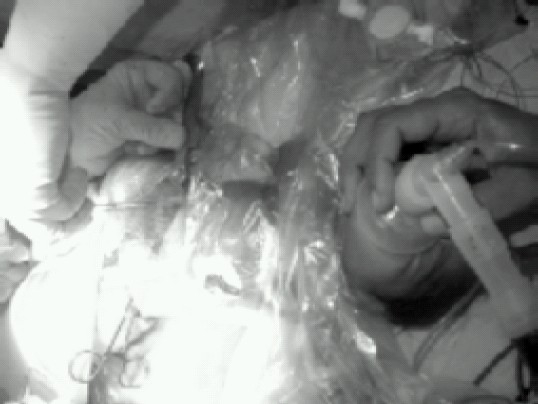
Neonate maintained on spontaneous ventilation

A 10-hour-old full term neonate, weighing 1.6 kg was posted for gastroschisis repair. On presentation his stomach, small and large intestine, gall bladder were lying outside the abdominal cavity with abdominal wall defect at the level of umbilicus. Neonate was moderately active with good cry. Pulse measured 130/minute; respiratory rate was 40/min. Cardiorespiratory system evaluation was normal. Biochemical investigations were also within normal limits. Preoperatively, 10% dextrose with added calcium gluconate (4 ml i.e. 36 mg elemental calcium/day) and potassium chloride (3.2 ml i.e. 6.4 meq/day) at 80 ml/kg/day was administered. Cardioscope, pulse oximeter, temperature probe and non invasive blood pressure cuff were attached. Atropine 0.1 mg was administered and the neonate was sedated with 1% sevoflurane by mask. Subarachnoid block was performed with neonate in left lateral position in L4-L5 interspace using 27G, 2 inches BD^®^ spinal needle. 0.5 mg 0.5% bupivacaine, Astra Zeneca^®^ + 0.5 mcg of fentanyl to make a total volume of 0.2 ml was administered using a 1 ml Dispovan^®^ tuberculin syringe (dead space 0.02 ml). Spinal needle was removed after 5 seconds of completion of drug injection to prevent loss of drug due to skin tracking. Subsequently caudal epidural was performed using 19G epidural needle (Portex^R^) and 21G catheter was threaded 8 cm inside epidural space with tip lying approximately at T8 level. The catheter was then tunneled away from the site of entry to avoid soiling (Fig 1). The entire procedure took 10 minutes. The onset of subarachnoid block was judged by lower extremity motor block and patient not crying on stretching of abdominal wall. Intraoperatively, the neonate maintained spontaneous respiration with supplemental oxygen at 2 liters/minute and 0.5% sevoflurane administered via J-R circuit (Fig 2). Intra operatively, Ringer lactate was infused at 10 ml/kg/hr. (Total intraoperative fluid administered 22 ml). Supplemental analgesia was given through epidural route 30 minutes after subarachnoid block with 2 ml of 0.25% bupivacaine. Primary closure of abdominal wall defect was completed in 80 minutes. Again 1 hour after 1^st^ epidural bolus dose, 0.2% bupivacaine 0.5 ml was repeated epidurally using a 1 ml tuberculin syringe(10 mg i.e.2 ml of 0.5% bupivacaine diluted to 5 ml with normal saline served as the stock solution). Total intraoperative dose of bupivacaine administered was 6.5mg.

Perioperative course was uneventful. Blood loss was minimal. Neonate was shifted to PACU and observed closely for signs of respiratory distress. Subsequent analgesic doses by epidural route were given at 6 hour intervals with 0.2% 0.5 ml bupivacaine. The epidural catheter was removed after 24 hours.

## Discussion

The practice of regional blocks for paediatric patients is well established. It has been proven to be an effective modality in paediatric surgery. It offers excellent relaxation without hypotension with minimal alterations in respiratory rate and heart rate.

Data since 1978 in Vermont infant spinal registry states that success rate for spinal anaesthesia has increased over the years to 97.4%^5^. They report a shorter induction time as compared to general anaesthesia and a low complication rate.

Management of high risk neonates undergoing major surgery presents various challenges. Respiratory status is often precarious. Respiratory depression and post operative apnoea are well known complications of general anaesthesia in this group of patients. Major abdominal surgeries under general anaesthesia often require post operative ventilatory support. Regional anaesthesia offers a chance to avoid these complications^6^.

Spinal anaesthesia to begin with gives a good dense block and good relaxation. It also gives a quiet child to insert the epidural catheter. Duration of subarachnoid block in neonates is short due to higher metabolic rate and greater anaesthetic absorption due to higher vascular supply to spine. For prolonged surgery and post operative analgesia combination of spinalepidural technique is therefore advantageous^4^,^7^. Epidural analgesia also avoids need for opioid analgesic injections in post operative period and the associated respiratory depression. Other advantages of regional anaesthesia include reduction of post operative stress response and decreased incidence of post operative hypoxemia and bradycardia.

Especially important in gastroschisis repair is relaxation of abdominal wall provided by the spinal which allows for optimal reduction. The total closure of abdominal wall defect would result in tightness during abdominal movements during spontaneous respiration. In spontaneously breathing neonate this respiratory embarrassment is easily evident which thereby allows surgeon to decide on the feasibility of primary closure^8^.

Both, continuous infusion and intermittent top-up doses of local anaesthetic via epidural route have been used in neonates, however keeping in view the variable pharmacokinetics of bupivacaine, intermittent top –ups are considered the better option^9^.

In conclusion, we report the safe administration of combined spinal epidural to a neonate for gastroschisis repair and recommend it as a suitable alternative to general anaesthesia.
